# EHMTI-0158. Positive allosteric modulators (PAMS) of the metabotropic glutamate receptor 2 (MGLUR2) are effective against nerve injury induced facial allodynia in rats

**DOI:** 10.1186/1129-2377-15-S1-F9

**Published:** 2014-09-18

**Authors:** C Horváth, K Galgóczy, K Kordás, A Kis-Varga, G Túrós, G Szabó

**Affiliations:** 1 Pharmacological and Drug Safety Research, Gedeon Richter Plc., Budapest, Hungary; 2 Medicinal Chemistry II., Gedeon Richter Plc., Budapest, Hungary

## 

Activation of mGluR2 is known to induce analgesic effect in animal pain models. mGluRs have been found in trigeminal ganglia and dura of rats.

Our aim was to examine effect of mGlu2 PAMS in facial allodynia model induced by partial ligation of infraorbital nerve (pIONL) in rats.

The two selective mGlu2 PAM compounds studied, JNJ-40068782 (JNJ), and Compound A (CA) of G.Richter have high in vitro potency with functional EC50 values on human mGlu2R of 8 nM and 39 nM, respectively.

Allodynia was measured by von Frey filaments at least ten days after pIONL. Gabapentin, a drug used in neuropathic pain, and sumatriptan, an anti-migraine drug significantly reduced allodynia.

Single p.o. dosing of CA produced dose-dependent reversal of allodynia with ED50=1.7 mg/kg and maximum effect of 92% at 10 mg/kg dose. In a 7-day repeated dose study with 0.5 mg/kg dose, a sustained, significant effect was seen. Similarly, no sign of tolerance was seen with 10-day repeated administration of JNJ at 30 mg/kg p.o. dose, selected based on acute dose-response study.

Potential side effects of both compounds were monitored by an automated behavioral observation system. In the above examined dose range and dosing regimens (acute and chronic) no considerable side effects were detected. No effect of CA on thermal or mechanical nociceptive threshold was seen up to 10 mg/kg dose.

Our results suggest potential utility of mGluR2 PAMs against trigeminal neuralgia or migraine. CA proved to be similarly effective but much more potent in vivo than JNJ.

